# Energy Restriction during Childhood and Early Adulthood and Ovarian Cancer Risk

**DOI:** 10.1371/journal.pone.0027960

**Published:** 2011-11-23

**Authors:** Leo J. Schouten, Boukje A. C. van Dijk, L. H. Lumey, R. Alexandra Goldbohm, Piet A. van den Brandt

**Affiliations:** 1 Department of Epidemiology, GROW – School for Oncology and Developmental Biology, Maastricht University, Maastricht, The Netherlands; 2 Comprehensive Cancer Centre Netherlands, Groningen, The Netherlands; 3 Department of Epidemiology, Mailman School of Public Health, Columbia University, New York, New York, United States of America; 4 Department of Life Style, TNO, Leiden, The Netherlands; University of Nebraska Medical Center, United States of America

## Abstract

Dietary energy restriction may protect against cancer. In parts of the Netherlands, mostly in larger cities, periods of chronically impaired nutrition and even severe famine (Hunger Winter 1944–1945) existed during the 1930s and World War II (1940–1945). We studied the association between energy restriction during childhood and early adulthood on the risk of ovarian cancer later in life. In 1986, the Netherlands Cohort Study was initiated. A self-administered questionnaire on dietary habits and other cancer risk factors was completed by 62,573 women aged 55–69 years at baseline. Follow-up for cancer was established by record linkage to the Netherlands Cancer Registry. After 16.3 years of follow-up, 364 invasive epithelial ovarian cancer cases and 2220 subcohort members (sampled from the total cohort directly after baseline) with complete information confounders were available for case-cohort analyses. In multivariable analysis, ovarian cancer risk was lower for participants with an unemployed father during the 1930s (Hazard Ratio (HR), 0.70; 95% Confidence Interval (CI), 0.47–1.06) compared to participants with an employed father as well as for participants living in a city during World War II (HR, 0.69; 95% CI, 0.54–0.90) compared to participants living in the country-side. Residence in a Western City during the famine (Hunger Winter) was not associated with a decreased risk. Our results show a relation between proxy variables for modest energy restriction over a longer period of time during childhood or early adulthood and a reduced ovarian cancer risk.

## Introduction

Ovarian cancer is the seventh most common malignancy among women living in the world, with approximately 225,000 newly diagnosed ovarian cancer cases and almost 140,000 deaths due to ovarian cancer in 2008. [1]

Oral contraceptive use, parity and tubal ligation have been associated with a reduced ovarian cancer risk. [2], [3] Height has been associated with an increased risk of ovarian cancer. [4] Increased risks have also been reported for postmenopausal hormone use, intake of lactose/galactose, overweight and smoking, but these factors are inconsistently associated with ovarian cancer. [2]

In animal studies an inverse association between energy restriction and cancer has been found for mice [5], while such an association was less consistent for breast cancer [6], [7], [8] and colorectal cancer [9], [10], [11], [12] in humans. Restriction of calories in chickens resulted in a near five-fold reduction in ovarian adenocarcinoma.[13] The associations between height [2], [4], [14] and age at menarche [2] and ovarian carcinoma suggest that exposures during childhood and early adulthood, such as energy restriction, may also play a role in the etiology of ovarian cancer.

In the Netherlands, periods of chronically impaired nutrition existed in parts of the population during the economic depression in the 1930s and the earlier years of World War II (1940–1944). Also, a substantial part of the population experienced a severe famine during World War II, the so-called Hunger winter (1944–1945), especially in the Western Cities of the country.

This unique setting provided the opportunity to study the association between energy restriction during childhood and early adulthood on the risk of ovarian cancer later in life. We evaluated this within the Netherlands cohort study on diet and cancer.

## Materials and Methods

### The Netherlands Cohort Study on diet and cancer (NLCS)

The NLCS started in September 1986, when 62,573 women, 55–69 years of age at baseline, were enrolled in the cohort. A detailed description of this prospective cohort study has been reported elsewhere. [15] Briefly, baseline exposure data were collected by means of a self-administered questionnaire. The questionnaire included questions on lifestyle and dietary factors and other risk factors for cancer.

For efficiency reasons, data processing and analysis were based on the case-cohort approach, in which the cases were enumerated for the entire cohort (providing the numerator information for estimating incidence rates), while the accumulated person-years for the entire cohort were estimated from a subcohort randomly sampled from the entire cohort at baseline (providing the denominator information for estimating incidence rates).[15]

### Ethics statement

On recruitment, participants were informed in writing of the details of the study and its objectives. In accordance with the regulations at that time, written informed consent was obtained when participants returned the completed baseline questionnaire. The Medical Ethics Committee of Maastricht University/ University Hospital Maastricht, the Netherlands, has approved the study.

### Follow-up

After 16.3 years of follow-up (until 31 December 2002), information regarding vital status was available for all 2,589 female subcohort members. After the exclusion of women who had reported a prevalent malignancy (other than skin cancer; n = 151) or to have undergone an oophorectomy (n = 32), 2,406 female subcohort members were available for analysis.

Incident cancer cases occurring in the entire cohort have been identified by record linkage to the Netherlands cancer registry and the national pathology register (PALGA). The method of record linkage has been described earlier.[16] The completeness of cancer follow-up has previously been estimated to be >96%.[17]

During 16.3 years of follow-up, 421 incident, microscopically confirmed, primary ovarian cancer cases (International Classification of Diseases for Oncology [ICD-O]-3: C56.9) were identified. After excluding non-epithelial tumors (N = 13) and borderline invasive tumors (N = 14), 394 invasive epithelial ovarian carcinoma cases remained eligible for analysis.

### Assessment of energy restriction

Individual food intake data of our participants during childhood and early adulthood was not available, therefore we used the employment status of the father during the Economic Depression (1932–1940), residence during the Second World War (1940–1944), and residence in the winter of 1944–1945 (Hunger winter) as proxy variables to classify participants into groups of energy restriction. These variables were used since the caloric intake was reported to be lower in the families of the unemployed during the years of the economic depression [18], [19], [20], lower in the cities during the War years [21], [22] and much lower in the Western part of the Netherlands during the hunger winter of 1944–1945 (although in rural areas the situation was somewhat better than in the cities). [23], [24] Employment status was categorized into participants whose father had a job during the years of the economic depression or worked intermittingly and cohort members with fathers without a job during these years. Residence during the War years was based on the question to list the last 4 residences before baseline of the study, which resulted in a classification into living in a city (defined as a town with at least 40,000 residents) or living in a rural area in 1942 (the midpoint of the War years 1940–1944). Residence during the Hunger winter was based on the reported residence during the winter of 1944–45 and classification into non-Western part of the country, Western rural area and Western city was performed. Eleven cities in the west of the country were considered famine cities based on the definition by Stein et al. [23]: Amsterdam, Rotterdam, The Hague, Utrecht, Zaandam, Hilversum, Amersfoort, Dordrecht, Vlaardingen/Schiedam, Delft and Leiden.

Participants reporting to have lived abroad during the War years or during the Hunger winter were excluded from the analyses (leaving 381 cases and 2293 subcohort members), since their reported whereabouts make it difficult to speculate on the degree of energy restriction experienced.

### Statistical analyses

In all models, age was included to adjust for the increasing cancer risk with age. Confounders were selected in two steps: some were chosen a priori to be included in the models, and other potential confounders were only included if they changed age-adjusted hazard ratios (HRs) of any of the exposure variables by more than ten percent. The a priori selected covariables were: use of oral contraceptives (ever versus never) and parity (continuous). As potential confounders we further investigated age at menarche (continuous), age at menopause (continuous), hysterectomy (possible/probable or no), height (continuous) and cigarette smoking (never, ex or current). None of the potential confounders did change the age-adjusted HRs by more than 10% and the final model included therefore age, parity and use of oral contraceptives. Cohort members with missing values on any of the confounders were excluded, leaving 364 cases and 2220 subcohort members. In an additional multivariable model, we mutually adjusted for all exposure variables for energy restriction. Height and age at menarche could be intermediate factors and were therefore investigated by comparing the multivariable model to the model including either height or age at menarche. Furthermore, we explored associations in 5-year age groups based on year of birth, because the timing of exposure in relation to a possible susceptible period in life, such as menarche, may be important.

HRs and corresponding 95% confidence intervals (95% CI) for ovarian carcinoma risk were estimated in age-adjusted and multivariable adjusted analyses using the Cox proportional hazards model [25], [26] processed with the STATA statistical software package (StataCorp, College Station, TX). Standard errors were estimated using the robust Huber-White sandwich estimator to account for additional variance introduced by sampling from the cohort [27]. The proportional hazards assumption was tested using the scaled Schoenfeld residuals [28], and found to be justified. Two-sides p values are reported throughout the article and were considered statistically significant at a p value <0.05.

## Results

The most frequent subtypes of ovarian cancer observed in this population were serous carcinoma (49%), mucinous carcinoma (9%), endometrioid carcinoma (9%) and adenocarcinoma – not otherwise specified (24%).

Descriptive data did not show large differences in the distribution over the categories of the exposure variables between the cases and the subcohort, although cases were on average taller (165.8 versus 165.3 cm) than subcohort members (table 1). The reported age at menopause was somewhat higher for cases than for subcohort members, while there was no difference in age at menarche. A higher percentage of subcohort members had ever used oral contraceptives. Subcohort members more often had a hysterectomy. Also, subcohort members more often had children.

**Table 1:** Descriptive data for cases and subcohort members, according to exposure variables and other potential risk factors for ovarian cancer, Netherlands Cohort Study on Diet and Cancer, 1986–2002.

		Cases		Subcohort	
		N	(%)	N	(%)
Total		364	(100.0%)	2220	(100.0%)
Father work during Economic Depression					
Yes		313	(86.0%)	1847	(83.2%)
No		28	(7.7%)	238	(10.7%)
Missing		23	(6.3%)	135	(6.1%)
Residence during World War II					
Country-side		154	(42.3%)	801	(42.3%)
City		124	(34.1%)	892	(34.1%)
Moved >4 times since 1942		41	(11.3%)	247	(11.3%)
Unknown		45	(12.4%)	280	(12.4%)
Residence during hunger winter					
Non-West		211	(56.0%)	1235	(55.6%)
Country-side West		47	(12.9%)	327	(14.7%)
City West		99	(27.2%)	611	(27.5%)
Unknown		7	(1.9%)	47	(2.1%)
Age	Mean (sd)	62.0	(4.3)	61.5	(4.3)
Height (cm)	Mean (sd)	165.8	(6.0)	165.3	(6.2)
Use of oral contraceptives					
Never		301	(82.7%)	1683	(75.8%)
Ever		63	(17.3%)	537	(24.2%)
Years of oral contraceptive use†	Mean (sd)	6.2	(4.9)	7.5	(5.4)
Hormone replacement therapy					
No		304	(87.4%)	1847	(87.5%)
Yes		44	(12.6%)	263	(12.5%)
Age at menarche	Mean (sd)	13.7	(1.8)	13.7	(1.8)
Age at menopause	Mean (sd)	49.3	(4.1)	48.6	(4.5)
Number of children					
Nulliparae		84	(23.1%)	406	(18.3%)
1 child		34	(9.3%)	182	(8.2%)
2 children		94	(25.8%)	486	(21.9%)
> = 3 children		152	(41.8%)	1146	(51.6%)
Hysterectomy					
No		329	(90.4%)	1845	(83.1%)
Possible / probable		35	(9.6%)	375	(16.9%)

† Only for women who ever used oral contraceptives.

Age-adjusted and multivariable adjusted hazard ratios are shown in table 2. Participants with an unemployed father during the Economic Depression had a non statistically significant lower risk of ovarian cancer (multivariable HR 0.70; 95% CI, 0.47–1.06) compared to participants with an employed father. After mutual adjustment for the other exposure variables, the HR was 0.63 (95% CI, 0.37–1.05).

**Table 2:** Age-adjusted and multivariable adjusted hazard rates for exposure factors for energy restriction, Netherlands Cohort Study on Diet and Cancer, 1986–2002.

	Cases	Person-years in subcohort	Age-adjusted		Multivariable adjusted#		Cases	Person-years in subcohort	Multivariable und mutually adjusted†	
	N	N	HR*	95% CI	HR	95% CI	N	N	HR	95% CI
										
Father work during Economic Depression										
Yes	313	27514	1	Ref	1	Ref	242	20891	1	Ref
No	28	3462	0.70	0.46–1.05	0.70	0.47–1.06	18	2613	0.63	0.37–1.05
										
Residence during World War II										
Country-side	154	11974	1	Ref	1	Ref	143	11226	1	Ref
City	124	13190	0.73	0.57–0.94	0.69	0.54–0.90	117	12279	0.72	0.53–0.99
										
Residence during Hunger winter										
Non-West	211	18445	1	Ref	1	Ref	157	13248	1	Ref
Country-side West	47	4854	0.86	0.61–1.24	0.88	0.63–1.20	37	3467	0.91	0.61–1.35
City West	99	9007	0.96	0.74–1.24	0.93	0.63–1.24	66	6790	0.97	0.68–1.39

*HR =  Hazard Rate; CI =  Confidence Interval.

# Multivariable adjusted for age, number of children (continuous) and use of oral contraceptives (never, ever).

† Multivariable adjusted for age, number of children (continuous), use of oral contraceptives (never, ever), for hysterectomy (no, possible/probable) and mutually adjusted for the other exposure factors in the table.

The risk of ovarian cancer was lower (HR 0.69; 95% CI, 0.54–0.90) for participants living in a city during World War II in comparison to participants living in the country-side. After mutual adjustment for the other exposure variables for energy restriction, the HR was 0.72, (95% CI, 0.53–0.99).

The risk of ovarian cancer for residents of a Western City during the Hunger Winter was 0.93 (95% CI, 0.63–1.24) compared to participants who lived in the non-West part of the country. After mutual adjustment for the other indicators for energy restriction, the HR was 0.97 (95% CI, 0.68–1.39).

When the analyses were restricted to serous ovarian cancer cases, results were very similar (data not shown).

In table 3, the results are shown stratified by the three 5-year birth cohorts. Because of the small numbers of cases with unemployed fathers in the cohorts, the risk estimates in each birth cohort are very unstable and mutually indistinguishable (*p* for interaction, 0.65). For individuals living in a city during World War II relative to those living in rural areas, estimates in each birth cohort were not different (*p* for interaction, 0.19). The risk estimates for Living in a Western City or Western rural area during the Hunger Winter of 1944–1945 were not consistent (*p* for interaction, 0.07). When the analyses were stratified according to age at diagnosis (before or after age 70), results were very similar (data not shown).

**Table 3:** Multivariable adjusted* hazard rates for exposure, stratified by 5-year age groups based on year of birth, Netherlands Cohort Study on Diet and Cancer, 1986–2002.

	Born between 1916 and September 1921				Born between September 1921 and September 1926				Born between September 1926 and 1931				p for interaction
Exposure	Cases	Person yrs	HR†	95% CI†	Cases	Person yrs	HR†	95% CI†	Cases	Person yrs	HR†	95% CI†	
Father work during Economic Depression													
*Age range in 1933*–*1934*	*12*–*18 yrs*				*7*–*13 yrs*				*2*–*8 yrs*				
Father had a job	100	7041	1	Ref	107	9371	1	Ref	106	11101	1	Ref	
Father had no job	8	1106	0.51	0.24–1.10	12	1087	0.93	0.48–1.77	8	1270	0.69	0.32–1.48	0.65
Residence during World War II													
*Age range in 1942*–*1943*	*21*–*27 yrs*				*16*–*22 yrs*				*11*–*17 yrs*				
Rural area	42	3056	1	Ref	55	4088	1	Ref	57	4830	1	Ref	
City	43	3839	0.76	0.48–1.22	39	4145	0.64	0.41–1.01	42	5206	0.68	0.44–1.05	0.19
Residence during Hunger winter^§^													
*Age range in 1944*–*1945*	*23*–*29 yrs*				*18*–*24 yrs*				*13*–*19 yrs*				
Non-West	58	4977	1	Ref	82	6122	1	Ref	71	7346	1	Ref	
Western rural area	20	1020	1.73	0.98–3.06	15	1732	0.66	0.36–1.19	12	2101	0.60	0.32–1.16	
Western city	37	2684	1.12	0.72–1.76	26	2910	0.64	0.40–1.04	36	3413	1.11	0.71–1.72	0.07

*Multivariable adjusted for age, number of children (continuous) and use of oral contraceptives (never, ever).

† HR =  Hazard rate; CI =  Confidence Interval.

The multivariable models were additionally adjusted for height and age at menarche which had little effect on the rate ratios (data not shown).

## Discussion

In this prospective cohort study, we used father's unemployment status during the Economic depression as an indicator of moderate energy restriction in childhood and city residence during World War II as indicators of moderate energy restriction during early adulthood. Both indicators were associated with a decrease in the risk of ovarian cancer. We found no association however between residence in a city in the Western Netherlands as an indicator of severe energy restriction and cancer risk.

Misclassification of energy restriction is possible since the exposure was measured indirectly by proxy variables. We believe these indicators of energy restriction can be reasonably adequate however, as previously discussed by Dirx et al. [7]. Female subcohort members were asked if they really had experienced hunger during the winter of 1944–1945. Seventy-five percent of the women living in a Western city replied affirmative. Of the 35% women who reported severe hunger during the Hunger Winter, 80% lived in a Western city during this winter. [7]

There are several publications that show the existence of a period of chronically impaired nutrition during the economic depression in the 1930s [18], [19], [20] and the earlier years of World War II in the Netherlands (1940–1944). [21], [22] During the Economic Depression, a relatively large proportion of the population was unemployed. Several surveys showed that there was little variation in the food pattern of the families with an unemployed father and that the energy intake was not at the same level as for the families with an employed father. [18], [19], [20] The nutrient composition of the diet was subject to change between 1941 and 1944: the contribution of carbohydrates to total energy intake increased, while the contribution of protein and fat decreased.[22] Although some authors reported that during the War years nutritional differences developed between cities and rural areas as a consequence of the poor availability of foods in the cities, [21], [22] more recent insights indicate that until the fall of 1944 the overall nutritional status of the population was adequate, in part because of effective food rationing. [24] It is therefore possible that the observed association with residence during World War II is coincidental and not causal.

A substantial part of the population experienced a severe famine during World War II, the so-called Hunger winter (1944–1945), especially in the Western part of the country. [24], [29] Contrary to expectation however we did not find an inverse association for participants living in a city in the West during the Hunger winter. This lack of association may be due to the fact that most women were no longer the teen years, which appears to be the most susceptible age range. Alternatively, the exposure period may have been too short. The two other exposure variables also seem to indicate that the inverse association may be stronger before a certain age. Our subgroup results give some suggestion of stronger effects of energy restriction during the teenage period (10 to 18 years of age) but the numbers are small.

Body Mass Index (BMI) reflects the balance of energy intake and expenditure. Energy restriction can be expected to cause a lower BMI. However, studies between body composition at different ages and ovarian cancer risk are not in agreement with our findings. In the Nurseś Health Study, birth weight was not, while body fatness at age 5 and 10 were inversely associated with ovarian cancer risk. [30] In the Nurseś Health Study II (NHSII), with fewer and younger cases, a non-statistically significant positive association was observed between body fatness at age 5 and 10 and ovarian cancer risk. [30] Body Mass Index (BMI) at age 18 or age 20 was not associated with ovarian cancer risk in a pooled analysis that included data from seven prospective cohort studies. [4] Information on body size during exposure periods is not available in our dataset and we could not investigate whether this was a confounder or an intermediate factor in our analysis.

Another explanation may be that not energy restriction, but nutrient composition is of importance. During the war years, especially the proportion of fat decreased.[22] A randomized controlled trial in postmenopausal women showed that restriction of the % energy (8–10% in the intervention group compared to the control group) from fat in the diet may reduce the risk of ovarian cancer risk by 17%, in the absence of substantial energy restriction.[31] This is in agreement with our observed association between indicators of energy restriction during the war years and a decreased ovarian cancer risk.

A lower risk of ovarian cancer would be in accordance with the theory postulated by Fathalla, who suggested that every ovulation causes trauma to the ovarian epithelium and stimulation of mitosis.[32] Less ovulations would therefore be associated with a lower ovarian cancer risk. Although age at menarche did not appear to be an intermediate factor in the current study, the attainment and maintenance of ovulatory cycles may be delayed under circumstances of dietary restriction as a result of low follicle stimulating hormone (FSH) and Luteinizing Hormone (LH) levels.[33] Also, it has been suggested that higher levels of gonadotropines, such as LH, are associated with a higher risk of ovarian cancer.[34] In an intervention study it was shown that caloric restriction was associated with a reduced secretion and pulsatility of LH in young women (mean age: 20.5 years), but not in adults (mean age: 28.7 years).[35]

In this large prospective cohort study, we were able to consider several potential confounders. Height and age of menarche do not appear to be intermediate factors however in the presumed relation of energy restriction during childhood and early adulthood and ovarian cancer. We believe that this study is the first to look at measures of modest energy restriction during childhood and early adulthood and the risk of ovarian cancer in later life and to find an inverse association. No relation was seen however between presumed severe energy restriction during the Hunger Winter and ovarian cancer risk, perhaps because the exposure period was much shorter (November 1944-May 1945). Other relevant studies include the study by Elias et al [36], who studied cancer incidence in another cohort of women that was exposed to the Dutch hunger winter, but the statistical power was too small to analyze the association with ovarian cancer. In a Danish record-linkage study of 2,151 women with anorexia nervosa (which could be regarded as an extreme form of caloric restriction) no cases of ovarian cancer were observed during follow-up.[37] Excluding cervical cancer, 2.4 cases of gynecological cancer were expected while no cases were observed. The power of this study, again, is too limited to draw conclusions.

Overall, using fathers occupation and wartime residence data as proxy measures for exposure, our study suggests that moderate energy restriction during adolescence was associated with a decrease in ovarian cancer risk. We found that our measure of more severe energy restriction during a shorter period of time was not. Other studies are needed to confirm these findings.
